# *Cryptococcus gattii* Meningitis Complicated by *Listeria monocytogenes* Infection

**DOI:** 10.3201/eid2209.160142

**Published:** 2016-09

**Authors:** Robert G. Deiss, Michael Bolaris, Angel Wang, Scott G. Filler

**Affiliations:** Uniformed Services University of the Health Sciences, Bethesda, Maryland, USA (R.G. Deiss);; Henry M. Jackson Foundation for the Advancement of Military Medicine, Bethesda (R.G. Deiss):; Naval Medical Center of San Diego, San Diego, California, USA (R.G. Deiss);; Harbor-UCLA Medical Center, Los Angeles, California, USA (M. Bolaris, A. Wang, S.G. Filler);; David Geffen School of Medicine at UCLA, Los Angeles (S.G. Filler)

**Keywords:** *Cryptococcus gattii*, *Listeria monocytogenes*, meningitis, cryptococcoma, corticosteroids, fungi, bacteria, co-infection

**To the Editor:** Among immunocompetent persons with cryptococcal disease, infection with a second organism is rare; all reported cases have involved concomitant mycobacterial infections (*1*). Immunosuppression is not a necessary precondition for infection with *Cryptococcus gattii* (*2*), and among immunocompetent persons, *C. gattii* infection confers high mortality rates: up to 24% according to a large case series (*3*). In addition, cryptococcomas are frequently observed in patients with *C. gattii*, as opposed to *C. neoformans*, infection, commonly necessitating longer courses of treatment. We report a fatal case of *C. gattii* and *Listeria monocytogenes* co-infection in an immunocompetent woman with cryptococcomas.

The patient was a previously healthy 23-year-old Hispanic woman who was hospitalized in 2009 after weeks of headache and recent-onset diplopia. Lumbar puncture revealed elevated opening pressure of 52 cm H_2_O; elevated leukocytes (1,030 cells/μL: 31% neutrophils, 55% lymphocytes, 14% monocytes); elevated protein concentration (117 g/L); and decreased glucose concentration (30 mg/dL). Cerebrospinal fluid (CSF) cryptococcal antigen (CrAg) titer was 1:64, and culture grew *C. gattii*. HIV antibody test result was negative. Magnetic resonance imaging of the brain demonstrated scattered enhancing round lesions within the cerebrum and cerebellum, consistent with cryptococcomas. The patient was prescribed intravenous amphotericin B (1 mg/kg/d) and intravenous flucytosine (2 g/6 h) (Table); after 5 days of therapy, culture of a repeat lumbar puncture sample was negative. The patient was then given intravenous liposomal amphotericin at 7 mg/kg, and after a 14-day induction period she was discharged with instructions to take fluconazole orally (400 mg 2×/d) and to continue amphotericin B infusions (3×/wk) (Table).

**Table:** Clinical events, management, and parameters for patient with *Cryptococcus gattii* meningitis complicated by *Listeria monocytogenes* infection*

Clinical event (day)	Therapy (days)	Opening pressure, cm H_2_O (day)	Leukocyte count, cells/μL (day)	Protein, g/L (day)	Glucose, g/L (day)	CrAg titer (day)	Culture result (day)
Days 1–15: induction therapy	AMB 1 mg/kg (1–4); 5FC 2 g q6h (1–14); L-AMB 7/mg/kg (5–14)	52 (1), 12 (12)	1,030 (1), 123 (12)	117 (1), 104 (12)	30 (1), 29 (12)	1:64 (1)	*Cryptococcus gattii* (1), negative (12)
Days 16–30: discharge, outpatient infusion, readmission	L-AMB 7/mg/kg M,W,F (15–22); FLZ 400 mg q12h (15–22); L-AMB 5 mg/kg (23–30); FLZ 600 mg q12h: (23–30); 5FC 3 g q6h (23–30)	46 (23)	111 (23)	81 (23)	34 (23)	1:8 (23)	Negative (23)
Days 31–45: inpatient therapy	L-AMB 5 mg/kg (31–45); FLZ 600 mg q12h (31–45); 5FC 3 g q6h (31–45)	44 (38)	17 (38)	66 (38)	64 (38)	NA	Negative (38)
Days 46–60: inpatient therapy	L-AMB 5 mg/kg (46–60); FLZ 600 mg q12h (46–60); DEX 2 mg q6h (46–60)	35 (48)	18 (48)	25 (48)	85 (48)	NA	Negative (48)
Days 61–75: discharge and outpatient infusion	L-AMB 5 mg/kg (61–65); FLZ 600 mg q12h (61–75); DEX 2 mg q8h (61–75); L-AMB 7/mg/kg M,W,F (66–75)	13 (63)	8 (63)	28 (63)	91 (63)	1:4 (63)	Negative (63)
Days 76–83: readmission/coma (80); death (83)	L–AMB 7/mg/kg M,W,F (76–79); DEX 2 mg q12h (76–79); FLZ 600 mg q12h (76–83); CRO 2 gm q12h (80–83); AMP 2 gm q4h (80–83); TMP/SMX 320–1,600 mg (2 double-strength tablets) q8h (80–83)	>55 (80)	1,010 (80)	258 (80)	17 (80)	1:4 (80)	*Listeria monocytogenes* (80)

*5FC, flucytosine; AMB, amphotericin B; AMP, ampicillin; CrAg, cryptococcal antigen; CRO, ceftriaxone; DEX, dexamethasone; F, Friday; FLZ, fluconazole; L-AMB, liposomal amphotericin; M, Monday; NA, not available; q, every; TMP/SMX, trimethoprim/sulfamethoxazole; W, Wednesday.

One week after hospital discharge, the patient experienced recurrent headache and low-grade fever and was readmitted. Repeat lumbar puncture indicated an opening pressure of 46 cm H_2_O but improvement of all other clinical parameters (Table). CSF CrAg titer was 1:8 and culture was negative. Repeat brain magnetic resonance images revealed no hydrocephalus, minimal edema, and decreased size and number of cryptococcomas. She was again given amphotericin B (5 mg/kg/d) and intravenous flucytosine (3 g/6 h) and fluconazole (600 mg/12 h). Placement of a ventricular-peritoneal shunt was deferred, and the patient required frequent lumbar punctures to relieve elevated intracranial pressure. After 3 weeks of therapy, she began taking oral dexamethasone (2 mg 4×/d) to reduce intracranial pressure and control symptoms consistent with immune reconstitution inflammatory syndrome. After 30 days of antifungal therapy during this second hospitalization, she experienced symptomatic improvement and was discharged with amphotericin B (5 mg/kg to be infused 3×/wk), fluconazole (600 mg 2×/d), and dexamethasone (tapering dosage).

Two weeks later (11 weeks after initial admission), she returned to the hospital with worsening headache and fever. Lumbar puncture demonstrated a leukocyte count of 1,010 cells/μL (74% neutrophils, 12% lymphocytes, 14% monocytes), glucose 17 mg/dL, protein 258 g/L, and an opening pressure of >55 cm H_2_O. CSF culture grew *L. monocytogenes*. The patient was prescribed ceftriaxone, ampicillin, and trimethoprim/sulfamethoxazole. Shortly after the lumbar puncture, she experienced status epilepticus and became comatose. Despite emergent craniotomy for relief of intracranial pressure, she remained comatose for several days; subsequently, supportive care was withdrawn and the patient died shortly thereafter.

This case highlights the difficulties of managing severe cryptococcal disease. This patient experienced headache over 3 months and symptom relapse during 10 weeks of anticryptococcal therapy. As was done in this case, practice guidelines recommend a longer duration of polyene antimycotic induction for patients with cryptococcomas than for those without (*4*), and longer courses of therapy are commonly described for infections caused by *C. gattii* than for those caused by *C. neoformans* (*5*). Corticosteroids are commonly used to treat immune reconstitution inflammatory syndrome associated with cryptococcal meningitis (*6*), although recently, they have been associated with adverse outcomes (*7*). As indicated by this case, corticosteroids remain a risk factor for secondary infection with several pathogens, including *Listeria*. No epidemiologic exposure to *Listeria* was identified for this patient.

*C. gattii* infection has been reported in 8 states, including California (*3*); we have found the pathogen in the soil south of Los Angeles, California, particularly in association with Canary Island pines and sweet gum trees (*8*). Some patients with *C. gattii* infection have autoantibodies to granulocyte–macrophage (GM) colony-stimulating factor (*9*). Although these autoantibodies have not been reported in patients with *Listeria* infections, susceptibility to infection caused by this bacterium is increased in GM–colony-stimulating factor –/– mice (*10*). Autoantibodies against GM–colony-stimulating factor or perhaps other cytokines might have impaired the patient’s host defense against these organisms; unfortunately, our report is limited by lack of serum for further testing.

This case demonstrates the difficulties of managing patients with *C. gattii* infection and an unusual co-infection with *L. monocytogenes*. Initiation of corticosteroids for the management of severe cryptococcal disease should be undertaken with caution. The differential diagnosis for worsening cryptococcal disease should include acute or subacute bacterial meningitis, particularly when the patient is receiving corticosteroids for the management of immune reconstitution inflammatory syndrome or associated complications.

## References

[1] Musabende M, Mukabatsinda C, Riviello ED, Ogbuagu O. Concurrent cryptococcal meningitis and disseminated tuberculosis occurring in an immunocompetent male. BMJ Case Rep. 2016;2016:bcr2015213380 . 10.1136/bcr-2015-213380 26917794 PMC4769440

[2] Chen SC; Australasian Society for Infectious Diseases (ASID) Mycoses Iterest Group. Cryptococcosis in Australasia and the treatment of cryptococcal and other fungal infections with liposomal amphotericin B. J Antimicrob Chemother. 2002;49(Suppl 1):57–61 . 10.1093/jac/49.suppl_1.57 11801583

[3] Harris JR, Lockhart SR, Sondermeyer G, Vugia DJ, Crist MB, D’Angelo MT, *Cryptococcus gattii* infections in multiple states outside the US Pacific Northwest. Emerg Infect Dis. 2013;19:1620–6. 10.3201/eid1910.130441 24050410 PMC3810751

[4] Perfect JR, Dismukes WE, Dromer F, Goldman DL, Graybill JR, Hamill RJ, Clinical practice guidelines for the management of cryptococcal disease: 2010 update by the Infectious Diseases Society of America. Clin Infect Dis. 2010;50:291–322. 10.1086/649858 20047480 PMC5826644

[5] Franco-Paredes C, Womack T, Bohlmeyer T, Sellers B, Hays A, Patel K, Management of *Cryptococcus gattii* meningoencephalitis. Lancet Infect Dis. 2015;15:348–55. 10.1016/S1473-3099(14)70945-4 25467646 PMC4481715

[6] Phillips P, Chapman K, Sharp M, Harrison P, Vortel J, Steiner T, Dexamethasone in *Cryptococcus gattii* central nervous system infection. Clin Infect Dis. 2009;49:591–5. 10.1086/603554 19589083

[7] Beardsley J, Wolbers M, Kibengo FM, Ggayi A-BM, Kamali A, Cuc NTK, Adjunctive dexamethasone in HIV-associated cryptococcal meningitis. N Engl J Med. 2016;374:542–54. 10.1056/NEJMoa1509024 26863355 PMC4778268

[8] Springer DJ, Billmyre RB, Filler EE, Voelz K, Pursall R, Mieczkowski PA, *Cryptococcus gattii* VGIII isolates causing infections in HIV/AIDS patients in southern California: identification of the local environmental source as arboreal. PLoS Pathog. 2014;10:e1004285. 10.1371/journal.ppat.1004285 25144534 PMC4140843

[9] Saijo T, Chen J, Chen SC-A, Rosen LB, Yi J, Sorrell TC, Anti-granulocyte-macrophage colony-stimulating factor autoantibodies are a risk factor for central nervous system infection by *Cryptococcus gattii* in otherwise immunocompetent patients. MBio. 2014;5:e00912–4. 10.1128/mBio.00912-14 24643864 PMC3967522

[10] Zhan Y, Lieschke GJ, Grail D, Dunn AR, Cheers C. Essential roles for granulocyte–macrophage colony-stimulating factor (GM-CSF) and G-CSF in the sustained hematopoietic response of *Listeria monocytogenes*–infected mice. Blood. 1998;91:863–9. 9446646

